# Initial partial response and stable disease according to RECIST indicate similar survival for chemotherapeutical patients with advanced non-small cell lung cancer

**DOI:** 10.1186/1471-2407-10-681

**Published:** 2010-12-14

**Authors:** Lijie He, Yuee Teng, Bo Jin, Mingfang Zhao, Ping Yu, Xuejun Hu, Jingdong Zhang, Songbai Li, Yaling Gao, Yunpeng Liu

**Affiliations:** 1 Department of Medical Oncology, The First Hospital, China Medical University, NO.155, North Nanjing Street, Heping District, Shenyang City, China; 2 Department of Respiratory Medicine, The First Hospital, China Medical University, NO.155, North Nanjing Street, Heping District, Shenyang City, China; 3 Department of Radiology, The First Hospital, China Medical University, NO.155, North Nanjing Street, Heping District, Shenyang City, China; 4 Department of Medical Oncology, The Oncology Hospital of Liaoning Province in China. NO. 44, Xiaoheyan Road, Dadong District, Shenyang City, China

## Abstract

**Background:**

Stable disease (SD) has ambiguous clinical significance for patients according to the dominant Response Evaluation Criteria in Solid Tumours (RECIST). The primary aims of the study were: (1) to clarify the clinical significance of SD by comparing the progression-free survival (PFS) of response and SD patients with advanced non-small cell lung cancer (NSCLC) after the first two courses of the standard first-line platinum-based chemotherapy; (2) to explore the relationship between the percentage change in tumour size and PFS among initial SD patients, in order to provide some guidance for clinicians in deciding continuation/termination of the current treatment at a relative early time.

**Methods:**

A total of 179 advanced NSCLC patients whose baseline CT image was available for review were included in the study. Another CT image was taken in the initial assessment after chemotherapy. A comparison of PFS between initial partial response (PR) and SD was used to determine whether significant differences exist. The relationship between the early percentage of change in tumour size of initial SD patients and their PFS was investigated. In addition, overall survival (OS), the secondary endpoint in this study, was investigated as well.

**Results:**

Patients with initial PR are not significantly distinguished from those with initial SD when their PFS is concerned (median PFS 249 days [95% confidence interval, 187-310 days] versus 220 days [95% confidence interval, 191-248 days], p > 0.05). Their median OS was 364 days (95% confidence interval, 275-452 days) for the initial PR patients versus 350 days (95% confidence interval, 293-406 days) for the initial SD patients, which suggests no significant difference as well p > 0.05). In addition, all the initial SD patients enjoyed similar PFS and OS.

**Conclusions:**

Initial PR and SD enjoy similar PFS and OS for patients with advanced NSCLC. Within the initial SD subgroup, different percentages of tumour shrinkage or increase undergo similar PFS and OS. RECIST remains a reliable norm in assessing the effectiveness of chemotherapy for patients with advanced NSCLC before functional assessment has been integrated into the criteria.

## Background

The change of tumour size is regarded as an objective indicator in assessing the efficiency of any anticancer therapy. Currently, the Response Evaluation Criteria in Solid Tumours (RECIST), which was established in 2000, and was revised in 2009, i.e. RECIST1.1, to replace the World Health Organization (WHO) criteria for tumour response evaluation, is widely used in evaluating the response to anticancer treatment. Using RECIST measurement criteria, patients in clinical treatments are stratified into one of four groups, i.e. complete response (CR), partial response (PR), stable disease (SD) and progressive disease (PD) on the basis of change in lesion size [1] derived from CT scans.

The purpose of stratification of patients into response categories is to acquire optimal treatment schemes. CR and PR indicate apparent decrease in tumour size. PD means a significant increase in tumour size or with the appearance of new lesions. SD is a relatively more complex category, and ranges from a minor decrease to a minor increase. It is generally accepted that a decrease in tumour size suggests effectiveness of the current therapy and an increase in tumour size indicates ineffectiveness. But when it comes to replacement of the current medical decision, only PD patients accept an alternative therapy in clinical practice. The maintenance of SD patients on their original treatment schemes is accepted as the norm in routine practice, although RECIST does not provide any reliable suggestion for clinicians to follow. Lara *et al*. share the same notion by claiming that initial disease control rate (DCR), including CR, PR and SD, is more powerful in predicting subsequent survival than the traditional tumour response rate alone (CR and PR). Non-progression of the disease is widely believed to have enjoyed survival benefits from the current treatment scheme [2]. Recently, an increasing number of experts propose that DCR should be used to predict survival [3].

The definition of response categories in RECIST is an arbitrary convention that is not based on any clinical data, for it has been adapted from the earlier WHO criteria on the assumption that the tumour is spherical model [4-7]. SD, with its complex components, has long been viewed as a controversial category with an equivocal result and its clinical significance is unclear. Many clinicians believe it is an arbitrary stratification of change in tumour size rather than a provision of any meaningful suggestion for clinical practice. Many physicians have tried to identify the clinical significance of SD and some claim that patients with initial SD after their first-line chemotherapy have poorer survival outcome and less symptomatic benefit than those with PR. Replacement of the current treatments is strongly recommended [8-12].

A further classification of SD research by W. De Roock et al. found that an initial relative decrease of more than 10% in tumour size at week 6 is associated with clinical benefit, and is regarded as a reliable cut-off point to predict the progression-free survival (PFS) and overall survival (OS) in chemorefractory metastatic colorectal cancer (cmCRC) patients treated with cetuximab (CTX) ± irinotecan (IRI). Thus, some SD patients have benefited from their treatment. This finding might be exploited in early discontinuation/crossover trials [13-15].

Especially, with the latest development of imaging techniques and the advent of targeted drugs, increasing numbers of patients achieve SD after biologic targeted therapies according to RECIST, whereas functional evaluation suggests that not all SD patients benefit directly from their treatments [16]. The biological behaviour of tumour cells is rather complicated; thus, further stratification of SD by tumour size is of great importance.

Chemotherapy is still the dominant treatment for advanced non-small cell lung cancer (NSCLC). However, the reality is that only a few patients with advanced NSCLC experience tumor decrease, i.e. "radiographic response" after standard first-line platinum-based chemotherapy and many more patients experience SD. So, it is very important to choose a suitable treatment regimen for advanced NSCLC patients who get SD in their initial assessment after the first two courses of first-line platinum-based chemotherapy. However, whether the different level of SD (the percentage of tumour shrinkage or increase) undergoes different PFS or even OS, whether all SD patients benefit from the original treatments is of great importance in current clinical practice. PFS is believed to be a rapid and accurate indicator for assessing the effectiveness of first-line treatment and predicting survival benefits. It can be used as a scale for retaining/replacing first-line treatments.

The main objectives of this study were: (1) to clarify the clinical significance of SD by comparing the PFS of PR and SD patients after the first two courses of chemotherapy; and (2) to explore the relationship between the change of tumour size, which ranges from minor decrease to minor increase, and their PFS among the achieved SD patients with advanced NSCLC according to RECIST in their initial assessment after the first two cycles of first-line platinum-based chemotherapy, to provide some guidance for clinical practice at a relatively early time.

## Methods

### Patient Selection

Between April 2007 and November 2009, 188 patients were enrolled and accepted medical treatment at two hospitals: the 1st Hospital of China Medical University and the Oncology Hospital of Liaoning Province in the northeast of China. Male and female patients in the age range 18 - 80 years were eligible for the research if they met the following criteria: histologically or cytologically confirmed with non-small cell lung cancer (NSCLC) that is unresectable, recurrent or metastatic; no previous adjuvant chemotherapy or recurrent metastatic NSCLC patients who have not received adjuvant chemotherapy for at least 6 months before the trial; at least one measurable tumour lesion not located in the locus that has received radiotherapy according to RECIST ver.1.1. Liver metastatic lesions that had received interventional therapy were not regarded as measurable lesions and hollow viscous lesions were not regarded as target lesions. The other requisites were: anticipated time of survival of at least three months; a desirable life exponent, i.e. 0 or 1 according to the Eastern Cooperative Oncology Group (ECOG) performance status; adequate renal, hepatic, cardiac and hematologic function. One week before enrolment in the study, the hematologic test should meet the following criteria: WBC ≥ 4.0 × 10^9^/L, ANC ≥ 2.0 × 10^9^/L, HB ≥ 10 g/dL, PLT ≥ 100 × 10^9^/L, TBI, ALT, AST, ALP and Cr counts less than 1.5 times the maximal peak, and TBI, ALT, AST, ALP less than 5 times the peak if liver metastasis occurs.

Patients were not enrolled in the study if they had only immeasurable lesions, such as malignant pleural effusion, ill-defined pulmonary densities and osseous metastasis, or if their only measurable lesion had received radiotherapy during the research; or they had brain metastases or meningeal metastases.

The study was totally in compliance with the Declaration of Helsinki, and was formally approved by the medical ethics committee of the First Hospital of China Medical University (No.200715). Each patient gave a written informed consent before any study-related procedures were performed.

### Treatments

All patients with advanced NSCLC were assigned to a combination of platinum-based chemotherapy, i.e. NP (vinorelbine, 25 mg/m^2 ^on days 1 and 8; cisplatin, 75-80 mg/m^2 ^on day 1), GP (gemcitabine, 1000 mg/m^2 ^on days 1 and 8; cisplatin, 75-80 mg/m^2 ^on day 1), PP (paclitaxel, 135-175 mg/m^2 ^on day 1; cisplatin, 75-80 mg/m^2 ^on day 1) or TP (docetaxel, 75 mg/m^2 ^on day 1; cisplatin, 75-80 mg/m^2 ^on day 1) treatment schemes according to their own conditions and wishes. Each patient received 4-6 courses of chemotherapy, and each course lasted 3 weeks. An assessment was made after every 2 courses of chemotherapy. Patients with PD were excluded from this group and received a different treatment regimen.

### Dosage Adjustment Scheme

Adverse events were graded according to the National Cancer Institute Common Toxicity Criteria (NCI-CTC) toxicity scale. If hematologic toxicity III or greater occurred, the dosage for the next cycle was decreased to 75-85% of the original dosage or the next course of treatment was postponed by 1 or 2 weeks. If hematologic toxicity III or greater remained, or a fever from combined severe infection and granulocytopenia IV occurred, the planned treatment was terminated. If non-hematologic toxicity (except trichomadesis) III or greater occurs, the dosage for the next course was adjusted to 75-85% of the original dosage. If cardiotoxicity II - IV occurred, treatment was discontinued. Throughout chemotherapy, adjuvant medicine such as antanacathartic, colony-stimulating factor and diphosphonate were given to reduce adverse events when necessary.

### Image Data Collection and Study

Each patient had a base-line check within one month before their first-line chemotherapeutic treatment started. To ensure the credibility of the study, images of measurable lesions were obtained by the qualified radiologists through CT or Spiro-CT scans. Subsequent images were obtained by the same radiologist after the first 2 courses of chemotherapy for each patient. To minimize variation between operators, all measurements of the images were done at the same anatomic level and orientation by an independent qualified radiologist. In contrast with the base-line images, any change in the size of target lesions was calculated according to RECIST ver.1.1. Patients with an increase of 20% or more in lesion size or those with new lesions were regarded as having PD and were excluded from the study. Patients with a change of lesion size ranging from an increase of <20% to a decrease of <30% and with no new lesion were stratified as having SD. Patients with a 30% or greater decrease in the target lesion were regarded as achieving PR. Patients with disappearance of the lesion were stratified as achieving CR. For those with SD, the percentage of change in tumour size has been recorded for further study.

### Follow-up Visit

A follow-up visit was given to each patient who achieved CR, PR and SD. The assessment process was repeated every 2 courses until the end of the chemotherapy, or the aggravation of the disease, and after that, every 2 months until the progression of the disease or death occurred. This kind of service could be provided to meet a patient's need at any time. The percentage of change in size of target tumours and the PFS were the main contents for a follow-up visit.

### Statistical Analysis

SPSS13.0 software was used for all statistical analysis. The correlation between the change in size of target lesions before and after the first two courses of chemotherapy and their PFS and OS were calculated. According to the RECIST ver.1.1, the sum of the longest dimension of all the target lesions was calculated for each patient to provide the baseline before treatment. The same measurements and calculation were done after the first two courses of chemotherapy to obtain the initial assessment sum. The change ratio of target lesions was calculated as:

(Assessment sum−Baseline sum)/Baseline sum

The primary endpoint was PFS, which is calculated from the date of first treatment to disease progression (local or metastatic) or death; and the secondary endpoint was OS, which is calculated from the date of first treatment to death resulting from any cause. The Kaplan-Meier curve was used to describe PFS and OS, and a log-rank test was used to compare the PFS and OS of each category defined according to the percentage reduction in tumour size i.e. ≥30% [PR], ≥25%, ≥20%, ≥15%, ≥10%, ≥5%, and ≥0%.The χ^2 ^test was used for qualitative data analysis, and the Kruskal-Wallis test or variance analysis was used to analyse the quantitative data. Variables included in the multivariate analysis were age, sex, grade of the disease, ECOG performance status, and weight loss. P < 0.05 was set as the level of statistical significance.

## Results

### Distribution of Response Categories and Patient Characteristics

**Figure 1:**
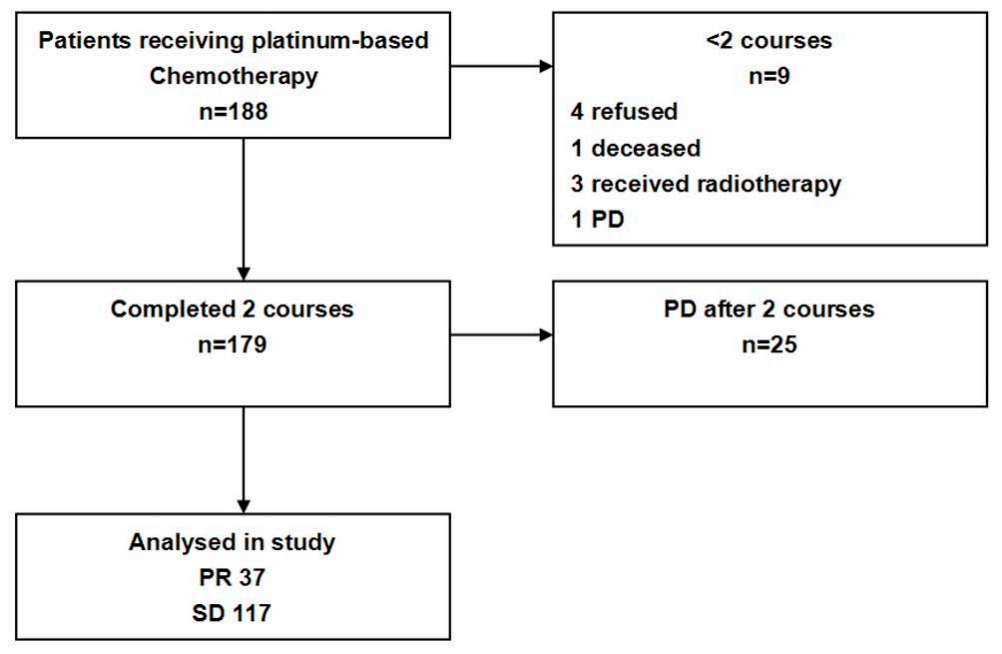
**Consort diagram of patient denominator**. PR partial response; SD stable disease; PD progressive disease.

Altogether, 188 patients were enrolled in the trial but 9 withdrew after completing their first cycle of chemotherapy. (Four refused to accept the treatment, one died from myocardial infarction, three received radiotherapy for the their target lesions, and one experienced PD with osseous metastasis.) The remaining 179 patients with inoperative NSCLC had the initial assessment after the first two courses of first-line platinum-based chemotherapy: 37 achieved PR and 117 were regarded as SD and these 154 patients (PR + SD) were eligible for the final analysis (Figure 1). The remaining 25 had PD and no CR according to RECIST. Table 1 summarizes the characteristics of the patients included in this analysis. The median age of the patients when enrolled into the study was 57 years (range 26 - 77 years), with 38% > 60 years. A total of 92 males (60%) were eligible for the analysis. The vast majority of patients (63%) had stage IV disease, 64 patients (42%) reported a weight loss of ≥5%, and the majority of patients had an ECOG performance status of 0 or 1.

**Table 1:** Patient Characteristics

Characteristics	Patients(PR + SD) (n = 154)
Age, years	
Median	57
Range	26-77
Sex	
Female	62
Male	92
Stage	
IIIB	57
IV	97
ECOG performance status	
0	84
1	70
Weight loss ≥5%	
Yes	64
No	90

PR Partial Response; SD Stable Disease; ECOG Eastern Cooperative Oncology Group.

### Distribution of Change in Tumour Size of Initial SD Patients

**Figure 2:**
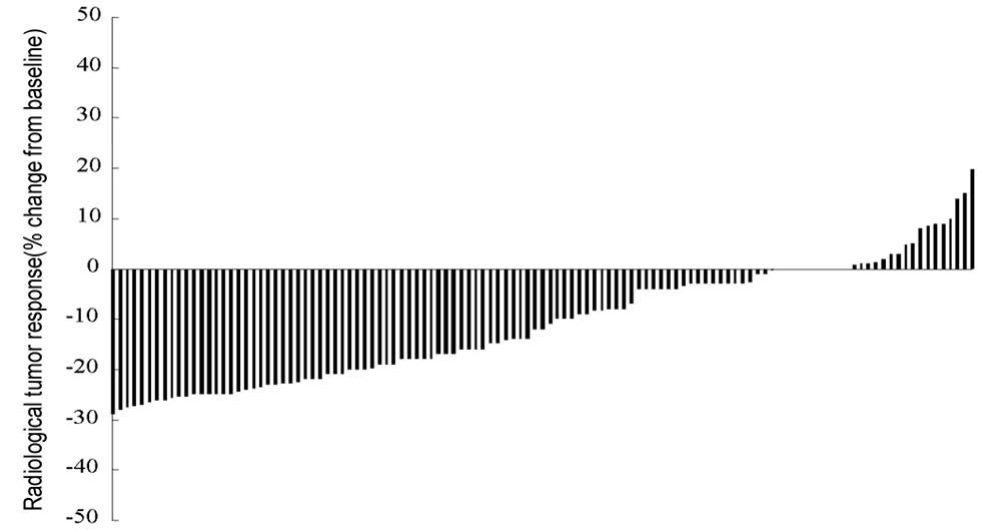
**Initial evaluation among SD patients in waterfall plot distribution (range -29% to +19.9%).** SD stable disease.

Among the patients with SD derived from the assessment after the first two courses of first-line platinum-based chemotherapy there was a decrease of tumour size of 25-30% for 17, 20-25% for 18, 15-20% for 16, 10-15% for 12 and 5-10% for 8 and 0-5% for 29. The increase of tumour size in the other patients was <20%. The result is depicted in waterfall plots (Figure 2).

### Comparison of PFS and OS of Patients with Initial PR and SD

**Figure 3:**
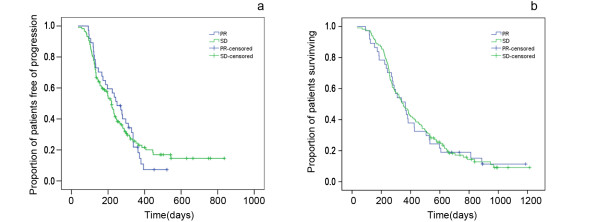
**Comparison of PFS (a) and OS (b) between PR and SD**. PR partial response; SD stable disease; PFS progression-free survival; OS overall survival.

The patients were divided into two subgroups (PR and SD) after two courses of treatment. Careful examination found no statistically significant difference of patient characteristics between the two groups, i.e. baseline characteristics were well balanced between PR and SD (Table 2). The median PFS was 249 days (95% confidence interval, 187-310 days) for the 37 patients with PR and 220 days (95% confidence interval, 191-248 days) for the 117 patients with SD. The log-rank test found no significant difference of PFS (P = 0.991) between the PR and SD subgroups (Figure 3a). The total follow-up time ranges from 2 months to 40 months, and by the end of the last follow-up time (Sept, 2010), 22 is still alive (5 PR, 17 SD). The median OS was 364 days (95% confidence interval, 275-452 days) for the PR patients and 350 days (95% confidence interval, 293-406 days) for the SD patients respectively. The log-rank test found no significant difference of OS (P = 0.861) between the PR and SD subgroups (Figure 3b).

**Table 2:** Patient Characteristics

	Response after 2 courses of platinum-based chemotherapy		
	
	PR (n = 37) (%)	SD (n = 117) (%)	P
Age, year, median	56	57	0.286
Sex, %			
Female	15(41%)	47(40%)	0.968
Male	22(59%)	70(60%)	
Stage, %			
IIIB	13(35%)	44(38%)	0.786
IV	24(65%)	73(62%)	
ECOG performance status, %			
0	25(68%)	59(50%)	0.068
1	12(32%)	58(50%)	
Weight loss ≥5%, %	14(38%)	50(43%)	0.598

PR Partial Response; SD Stable Disease; ECOG Eastern Cooperative Oncology Group.

### Relationship between Change in Target Lesion Size and PFS and OS among Initial SD Patients

**Figure 4:**
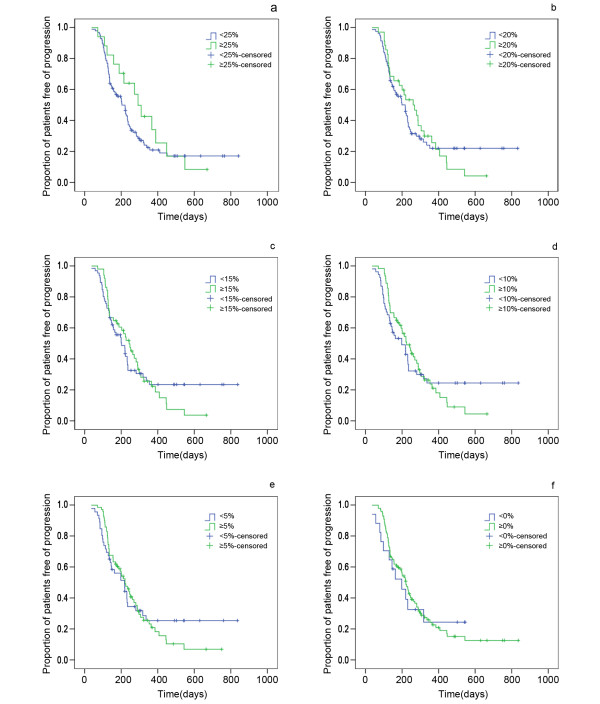
**Relationship between the changing ratio in size of target lesion and PSF among SD patients**. Comparison of PFS of SD patients stratified by a reduction in target lesion size of (a) 25%, (b) 20%, (c) 15%, (d) 10%, (e) 5%. and (f) 0%. SD stable disease; PFS progression-free survival.

**Figure 5:**
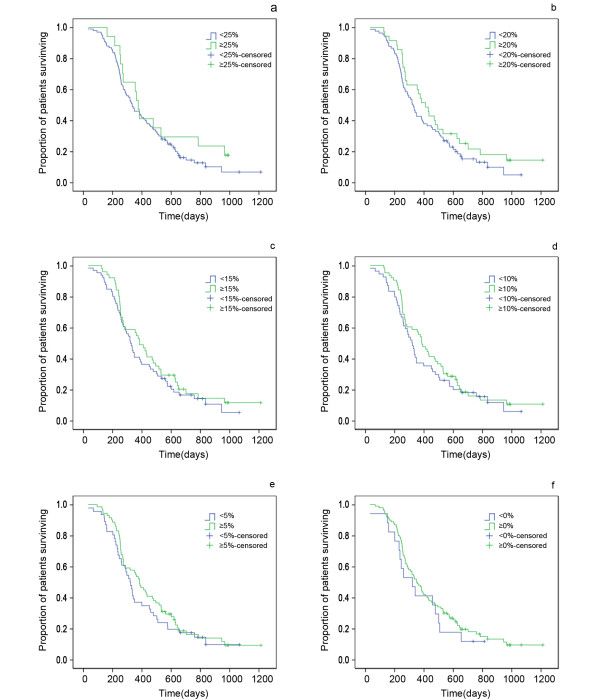
**Relationship between the changing ratio in size of target lesion and OS among SD patients**. Comparison of OS of SD patients stratified by a reduction in target lesion size of (a) 25%, (b) 20%, (c) 15%, (d) 10%, (e) 5%. and (f) 0%. SD stable disease; OS overall survival.

The response rate varied significantly according to the definition of "response rate" (Table 3). The median PFS of patients with ≥25%, ≥20%, ≥15%, ≥10%, ≥5% or ≥0% decrease in tumour size was 289 days, 270 days, 242 days, 225 days, 221 days or 221 days, respectively; the median OS was 377 days, 412 days, 385 days, 387 days, 385 days and 350 days, respectively. The P value associated with the log-rank test comparing the PFS and OS of responding patients with that of non-responding patients using different definitions of response are shown in Table 3 and Figure 4 and Figure 5. As indicated, no significantly better cut-off point, i.e. optimal percentage of decrease in tumour size, was found in predicting the PFS and OS of the patients with SD. In addition, among SD patients, those with an initial decrease of 30-20% in tumour size were not significantly distinguishable (P > 0.05) from those with an initial increase.

**Table 3:** Variable Response Criteria

Definition of responder	Group	No	Median PFS(95%CI)	P	**Dead No**.	Median OS(95%CI)	P
≥25% Reduction in tumour	Nonresponder	100	218(176-259)	0.319	86	335(261-408)	0.240
	Responder	17	289(225-352)		14	377(338-415)	
≥20% Reduction in tumour	Nonresponder	82	218(171-264)	0.874	71	323(276-369)	0.168
	Responder	35	270(181-358)		29	412(328-495)	
≥15% Reduction in tumour	Nonresponder	66	200(149-250)	0.937	57	323(279-366)	0.323
	Responder	51	242(190-294)		43	385(309-460)	
≥10% Reduction in tumour	Nonresponder	54	198(127-268)	0.948	47	320(268-371)	0.334
	Responder	63	225(176-273)		53	387(320-453)	
≥5% Reduction in tumour	Nonresponder	46	218(147-289)	0.789	40	320(272-367)	0.362
	Responder	71	221(177-265)		60	385(317-452)	
≥0% Reduction in tumour	Nonresponder	17	198(105-291)	0.913	15	323(195-450)	0.448
	Responder	100	221(193-249)		85	350(286-413)	

95% CI indicates 95% Confidence Interval; PFS Progression-free Survival; OS Overall survival.

Multivariate analysis included factors such as sex, age, weight loss, ECOG performance status, and stage of disease, which might have a potential effect on prognosis of NSCLC patients. Table 4 indicates that ECOG 1 and weight loss of ≥5% was relatively worse for predicting PFS (P < 0.05). Cox regression analysis showed that other prognostic factors like age, sex, stage of disease, the percentage of change in target lesion size of patients with SD derived from the initial assessment after the first two courses of chemotherapy are not associated with PFS (P > 0.05)

**Table 4:** Multivariate Analysis

Variable	Adverse covariate	Relative risk	95% CI	P
ECOG	1	2.757	1.162-6.546	0.021
Weight loss	≥5%	2.585	1.107-6.035	0.028

95% CI indicates 95% Confidence Interval; ECOG Eastern Cooperative Oncology Group.

## Discussion

OS has long been considered to be the most important indicator of clinical efficacy endpoints for any treatment regimen. However, OS requires prolonged follow-up, and is often affected significantly by other factors, such as the effects of second-line and subsequent therapies and so it cannot be used as a scale for adjusting first-line treatments. PFS, a biological indicator, can be applied to measure tumour burden directly. Especially, PFS can swiftly and accurately indicate the survival benefit of the first-line therapy, which enables clinicians to modify their treatment projects as soon as possible. Recent research has found that PFS is correlated positively with OS and it has been accepted as a surrogate for OS in advanced cancer [17-22].

RECIST, which is based on morphological features of the tumour, has been adopted as a norm for clinical trials. According to RECIST, patients who demonstrate a response often continue with their treatment protocols, and termination of the original therapeutic regimen and replacement with another has been considered for those who do not respond. In clinical practice, however, it is common to find that some patients demonstrate a dramatic response to chemotherapy, but they might not have any survival advantage when compared with the patients who do not respond [23,24]. Other patients who demonstrate SD or a minimal disease progression might end up with extended long-term survival. Focusing on this phenomenon, Katherine R et al. reached the conclusion that in their study of patients with advanced NSCLC there was no significant difference between the initial response categories of PR and SD with respect to overall survival [25]. In addition, Lara et al. suggested that SD enjoys equal survival significance with CR and PR. They claim that DCR, composed of CR, PR and SD, is more powerful than response i.e. CR and PR alone in predicting the overall survival of patients with advanced NSCLC [2]. Some experts suggest that SD might represent a potential survival benefit of chemotherapy. Therefore, the distinction between SD and PR might be of no practical importance [26,27].

Similar to the studies mentioned above, the results of the current study suggest that tumour shrinkage in the CT image derived from the initial treatment-assessment after the first 2 courses of first-line chemotherapy, i.e. early radiographic response in patients with advanced NSCLC is not positively related with their PFS and OS, thus, it cannot predict the outcome of chemotherapy. That is to say, patients with PR and SD derived from their initial assessment have similar PFS and OS. So, PR and SD enjoy the same clinical significance in predicting PFS or even OS according to RECIST. It is clear that patients with SD have also benefited from the continuation of the original treatment. The creative point is that this prospective study adopts treatment schemes with similar effectiveness to reduce any possible bias, and PFS as the end point, which can reflect effectiveness of their first-line treatment at a relatively early time, thus early adjustment of treatment is likely to be adopted.

SD is often used as a decisive point in treatment management in clinical research, but it is regarded as a category with much controversy. Continuation of the original treatment for the NSCLC patients with initial SD has been generally adopted. But the notion that prognosis of SD patients varies greatly due to the complexity of SD should be taken into careful consideration. Thus, a suggestion for further classification of SD is needed urgently for an alternative therapy at an early time. Certain further stratification of SD studies claim that an initial decrease in tumour size has little to do with overall survival, and no particular percentage of reduction in tumour size has been found to be correlated optimally with survival for patients with advanced NSCLC [25]. However, those studies do have several limitations, such as variation of treatment and their endpoint OS requires a long period of follow-up. Subsequent treatments might seriously interfere with OS, which cannot be regarded as evidence to guide early adjustment of first-line therapy. The current study uses similar treatment schemes and PFS as the primary end point. By further classifying SD patients, this study indicates that no optimal percentage change in tumour size associated with PFS exists in patients with NSCLC. All SD patients enjoy similar PFS and OS, which implies that they might benefit from their original treatments. This study further verifies that the current clinical norm of continuation of treatments in patients with NSCLC who have SD after two cycles of first-line platinum-based chemotherapy is the standard treatment.

As noted above, this study is based solely on morphological features, i.e. tumour size, which cannot reflect tumour cell viability in advanced NSCLC precisely. A study conducted by Liu-Jarin et al. also supports the notion that no correlation between tumour viability and radiographic response has been found after examination of the histological appearance of tumours after neoadjuvant therapy [28]. Since lung cancers are typically heterogeneous, composed of both cancer cells and a host inflammatory response in various proportions. Measuring tumour size by anatomic imaging studies alone provides only a macroscopic description of the lesion [29]. It is hard, sometimes even unlikely, to distinguish tumour from inflammatory response by a chest CT scan. For instance, a reduction in tumour cells but an increase in inflammatory component might seem equal to an increase in tumour cells but a decrease in inflammatory component as judged from early radiographic response alone. A similar phenomenon has been noted in the evaluation of gastrointestinal stromal cell tumours (GISTs). The treatments of GISTs with chemotherapeutic agents rarely results in a reduction of overall tumour size, but decreases the tumour cell burden, which is undetectable by macroscopic measurement alone [30].

Histological studies of treated NSCLC have demonstrated that the percentage of residual viable tumour cells is a reliable predictor of survival, and that many tumours that exhibit little change in overall size might actually have regressed significantly [31,32]. Recent imaging developments based on both tumour morphology and tumour function make correct, precise, and reproducible measurement available. In addition, it has been proved that functional response appears relatively earlier than radiographic response [33-35].

RECIST cannot accurately reflect the change of viability of a tumour cell. This is especially true with the occurrence of targeted therapy. Many studies indicate that after functional evaluation, some initial SD patients benefit greatly from their treatments, while some others should use an alternative therapy as early as possible. Just as Choi et al. suggest in their study that more sensitive criteria, a 10% decrease in size or a 15% decrease in density on contrast-enhanced CT, have been shown to be more accurate in the assessment of treatment efficacy and the prediction of survival in patients with GIST [16]. The application of PET, which can predict survival [36], to the initial SD subgroup might attain a functional response rather than an anatomical response. PET can provide some early guidance in clinical practice. Some published data indicate that the percentage [18F]fluorodeoxyglucose uptake after one and three courses of induction chemotherapy predicts survival in stage IIIA-N2 patients [37]. Still, integration of tumour biomarkers into standard response criteria might be useful in the evaluation of NSCLC because tumour biomarkers have been proven meaningful in determining tumour biology, viability in NSCLC, and predicting outcome in patients with early stage lung cancer [38,39]. Future studies could integrate functional evaluation into the initial SD subgroup, thus might provide some early guidance on continuation/replacement of chemotherapy. It might be more important to individualize treatment for initial SD patients with advanced NSCLC as early as possible to optimize the effectiveness of the treatment.

## Conclusion

Firstly, non-progression, i.e. PR, SD derived from the initial radiographic response after the first two courses of platinum-based chemotherapy enjoys similar PFS and OS for patients with advanced NSCLC; secondly, focused on the SD subgroup, different levels of SD (different percentages of tumour shrinkage or increase) undergo similar PFS and OS; lastly, RECIST remains a reliable norm in assessing the effectiveness of chemotherapy for patients with advanced NSCLC and instructing clinical medication before any functional assessment has been imported into the criteria. Hopefully, further studies will integrate functional assessment, such as contrast-enhanced CT and PET/CT, tumour biomarkers into the RECIST criteria, thus are likely to create a more reliable response criteria for advanced NSCLC in the future.

## Competing interests

The authors declare that they have no competing interests.

## Authors' contributions

LH conceived of the study, participated in design and prepare the manuscript, YT participated in the design of the study, BJ participated in the design and performed the statistical analysis, MZ participated in the design, PY participated in the design, XH participated in the design, JZ participated in the design, SL participated in the data collection, YG participated in the data collection, YL conceived of the study, participated in design and coordination. All authors read and approved the final manuscript.

## Pre-publication history

The pre-publication history for this paper can be accessed here:

http://www.biomedcentral.com/1471-2407/10/681/prepub
